# Optimization of Kernel Type and Sharpness Level Improves Objective and Subjective Image Quality for High-Pitch Photon Counting Coronary CT Angiography

**DOI:** 10.3390/diagnostics13111937

**Published:** 2023-06-01

**Authors:** Yang Yang, Nicola Fink, Tilman Emrich, Dirk Graafen, Rosa Richter, Stefanie Bockius, Elias V. Wolf, Gerald Laux, Larissa Kavermann, Lukas Müller, Michaela Hell, Moritz C. Halfmann

**Affiliations:** 1 Department of Diagnostic and Interventional Radiology, University Medical Center Mainz, Langenbeckstrasse 1, 55131 Mainz, Germany; yang.yang@unimedizin-mainz.de (Y.Y.); moritz.halfmann@unimedizin-mainz.de (M.C.H.); 2 Division of Cardiovascular Imaging, Department of Radiology and Radiological Science, Medical University of South Carolina, 25 Courtenay Dr, Charleston, SC 29425, USA; 3 Department of Radiology, University Hospital, LMU Munich, 81377 Munich, Germany; 4 Department of Cardiology, University Medical Center Mainz, Langenbeckstrasse 1, 55131 Mainz, Germany

**Keywords:** photon-counting detector CT, coronary CT angiography, high-pitch Flash mode, reconstruction kernels, image quality

## Abstract

(1) Background: Photon-counting detector (PCD) CT offers a wide variety of kernels and sharpness levels for image reconstruction. The aim of this retrospective study was to determine optimal settings for coronary CT angiography (CCTA). (2) Methods: Thirty patients (eight female, mean age 63 ± 13 years) underwent PCD-CCTA in a high-pitch mode. Images were reconstructed using three different kernels and four sharpness levels (Br36/40/44/48, Bv36/40/44/48, and Qr36/40/44/48). To analyze objective image quality, the attenuation, image noise, contrast-to-noise ratio (CNR), and vessel sharpness were quantified in proximal and distal coronaries. For subjective image quality, two blinded readers assessed image noise, visually sharp reproduction of coronaries, and the overall image quality using a five-point Likert scale. (3) Results: Attenuation, image noise, CNR, and vessel sharpness significantly differed across kernels (all *p* < 0.001), with the Br-kernel reaching the highest attenuation. With increasing kernel sharpness, image noise and vessel sharpness increased, whereas CNR continuously decreased. Reconstruction with Br-kernel generally had the highest CNR (Br > Bv > Qr), except Bv-kernel had a superior CNR at sharpness level 40. Bv-kernel had significantly higher vessel sharpness than Br- and Qr-kernel (*p* < 0.001). Subjective image quality was rated best for kernels Bv40 and Bv36, followed by Br36 and Qr36. (4) Conclusion: Reconstructions with kernel Bv40 are beneficial to achieve optimal image quality in spectral high-pitch CCTA using PCD-CT.

## 1. Introduction

Coronary artery disease (CAD) is the most common heart disease and the leading cause of mortality worldwide [1]. Due to its excellent sensitivity and negative predictive value for the diagnosis and exclusion of CAD, coronary computed tomography angiography (CCTA) has become an important part of diagnostic evaluations [2,3]. The results of several large randomized controlled trials have established CCTA as the first-line test for the assessment of patients with suspected CAD, particularly patients at low-to-intermediate risk of CAD [4,5,6].

Recently, the first-generation dual-source photon-counting detector (PCD) computed tomography (CT) was introduced and approved for routine clinical use. In contrast to conventional energy-integrating detector CT (EID-CT), PCD-CT counts and directly converts incoming photons into an electronic signal using semiconductor crystals. This detector enables the measurement of single photons and allows the registration of spectral information depending on the photon’s energy [7,8,9]. Therefore, compared to EID-CT, PCD-CT has several advantages, such as higher dose efficiency, higher spatial resolution, increased contrast-to-noise ratio (CNR), and higher image quality [10,11,12,13,14], and has promise to significantly improve cardiovascular imaging. In this context, preclinical phantom studies have already demonstrated that PCD-CT-derived CCTA scans have lower image noise, improved spatial resolution, and superior lipid core detectability, in comparison with EID-CT [15]. In addition, the first in vivo studies revealed improved visualization of calcified coronaries using ultra-high-resolution scanning [16], as well as higher image quality and diagnostic confidence compared to EID-CT when using PCD-CT for CCTA acquisition [17].

However, to maximize this new technology’s potential, the optimal settings for different indications need to be evaluated. Despite the knowledge that image reconstruction parameters, such as convolution kernels and iterative reconstruction, relevantly influence image quality in EID-CT [18], little is currently known about their influence in PCD-CT, especially for reconstruction of spectral data derived by high-pitch scans to calculate virtual monoenergetic images (VMIs).

Thus, the purpose of this study was to evaluate the objective and subjective image quality of high-pitch, spectral CCTA in PCD-CT to determine the optimal reconstruction kernel and sharpness level.

## 2. Materials and Methods

### 2.1. Study Population

This retrospective, single-center study was approved by the institutional ethics committee approval (Reg. Nr. 2022-16359) and performed in compliance with the Declaration of Helsinki.

Between February 2022 and March 2022, 34 patients who underwent CCTA were identified based on the following inclusion criteria: (1) CCTA was indicated to exclude or determine CAD, and (2) CCTA was performed with high-pitch Flash mode. Four patients with (1) non-evaluable coronary arteries or (2) contraindication against nitroglycerin were excluded. Thus, a total of 30 patients were included in this study. To accommodate for the requirements of the Coronary Artery Disease—Reporting and Data System (CAD-RADS [19]) classification system and thereby represent clinical reality, only segments of vessels with a diameter of ≥2 mm were evaluated in further analyses.

### 2.2. Data Acquisition

All CCTA scans were performed on a first-generation, dual-source CT scanner (NAEOTOM Alpha, Siemens Healthineers, Forchheim, Germany) containing two photon-counting, cadmium telluride detectors. Scans were acquired at 120 kVp with automated tube current modulation (CARE Dose4D) in the ‘Flash-QuantumPlus’ mode (pitch factor 3.2, gantry rotation time 0.25 s) with a detector collimation of 144 × 0.4 mm.

In the absence of contraindications and depending on the heart rate, patients were administered 0.8 mg nitroglycerin sublingually and a maximum of 10 mg intravenous metoprolol prior to the examination. All patients received a dual-bolus injection consisting of 15 mL iodinated contrast agent (Ultravist 370 mg iodine/mL, Bayer Healthcare, Berlin, Germany) for the test bolus and 75 mL for the main bolus. The ECG-pulsing window was set in diastole with start at 60% of the R-R interval.

### 2.3. Image Reconstruction

CCTA images were reconstructed on the scanner platform using three different kernels and four sharpness levels, resulting in a total of 12 reconstructions per CT scan: Body regular (Br) 36/40/44/48, Body vascular (Bv) 36/40/44/48, and Quantum regular (Qr) 36/40/44/48. The image slice thickness was set to 0.4 mm with an increment of 0.2 mm. Quantum iterative reconstruction (QIR) was used with a maximum strength level of 3 at the time of the investigation. Per vendor recommendation, VMIs were reconstructed at 55 kilo-electronvolts (keV) as standard output of the PCD-CT.

### 2.4. Objective Image Quality

All measurements were performed by two independent readers (MCH and YY, with 3 and 10 years of experience in cardiovascular CT, respectively).

Mean attenuation values were obtained by placing eight circular regions of interest (ROIs) at the following locations: left main stem (LM), proximal and distal regions of right coronary artery (RCA), left anterior descending (LAD) and left circumflex artery (LCX), and pericoronary fat. All ROIs were as large as possible while carefully avoiding inclusion of adjacent structures.

To ensure reliable results, the image processing and analysis software ImageJ (version 1.53) was used for the measurements [20]. ROIs were defined in one of the 12 reconstructed images and stored in the program’s ROI manager. A macro was created to automatically derive the mean attenuation values of the ROIs with identical position and size for all 12 reconstructions.

The attenuation value was expressed in Hounsfield units (HU). Image noise was determined as the standard deviation (SD) of attenuation measured in the ROIs. The contrast-to-noise ratio (CNR) were calculated using the following formulas:

CNR = (HU(coronary) − HU(fat))/(SD(coronary))

**Figure 1:**
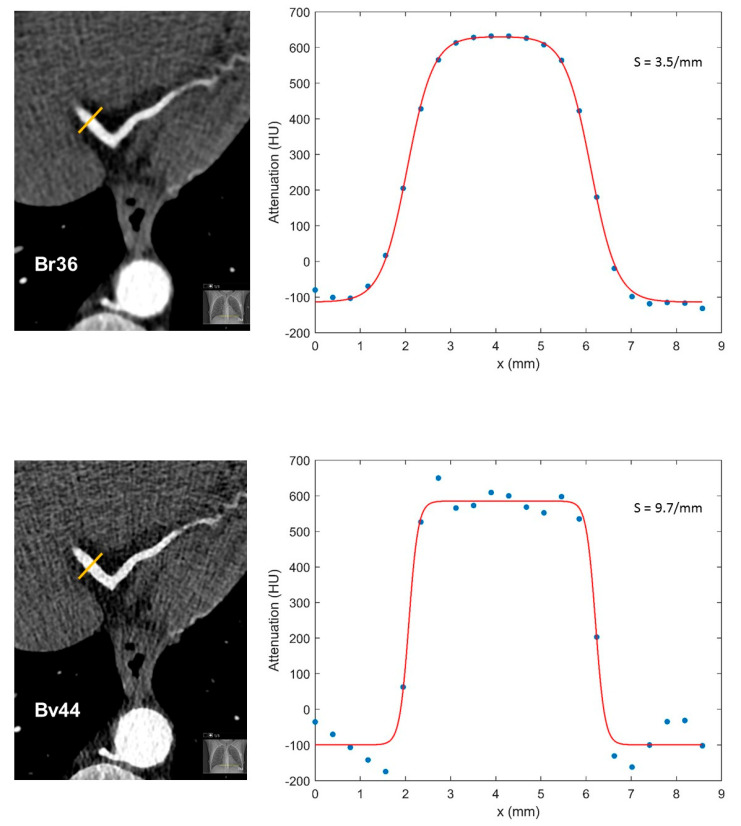
Example of vessel sharpness measurements. Line profiles of distal right coronary artery and corresponding double sigmoid fit functions with images reconstructed using kernels Br36 (**top**) and Bv44 (**bottom**). A considerably steeper slope, which presents a sharper vessel wall, can be observed in the reconstruction with the Bv44 kernel, S = 9.7/mm (**bottom**), compared to the Br36 kernel, S = 3.5/mm (**top**).

ImageJ was also used to analyze the sharpness of vessel borders by defining attenuation values along defined profiles. For each reconstructed image, a total of six profiles were placed perpendicular to the border of the coronary artery at the following locations: proximal and distal regions of the RCA, LAD, and LCX. Detected attenuation values were fitted with double sigmoid functions using the computing platform MATLAB (version R2021b, The MathWorks, Inc., Natick, MA, USA) according to the double sigmoid function:

**S**_double_ (x) = b + A × (1/((1 + e^(−s(x−x_1))^)) − 1/((1 + e^(−s(x−x_2))^)))

where the parameter **S** represents the border sharpness of the coronary artery. Examples of the double sigmoid functions with different sharpness parameters are shown in Figure 1.

### 2.5. Subjective Image Quality

Subjective image quality was independently assessed by the same two readers, who were blinded to the kernel types and sharpness levels. Image analysis was performed using the in-house picture archiving and communication system (Sectra, Linköping, Sweden). For each patient, all 12 reconstructed images (Br36/40/44/48, Bv36/40/44/48, and Qr36/40/44/48) were presented in a randomly ordered 3 × 4 side-by-side arrangement. Images were initially shown at predefined window settings (width: 900 HU; level: 250 HU), and manual adjustments from the readers were allowed according to personal preference.

Overall image quality, subjective image noise, and visually sharp reproduction of coronaries were assessed using a 5-point Likert scale. For overall image quality, the 5-point Likert scale consisting of the following categories was used: 1 = very poor; 2 = poor; 3 = moderate; 4 = good; 5 = excellent. Subjective image noise was assessed using the following categories: 1 = very strong image noise; 2 = strong image noise; 3 = moderate image noise; 4 = little image noise; 5 = no / very little image noise. Visually sharp reproduction of coronaries was analyzed as follows: 1 = unacceptable, nondiagnostic image quality; 2 = suboptimal image quality; 3 = acceptable visually sharp reproduction of coronaries; 4 = good and above average image quality; 5 = excellent image quality [16,17]. Exemplary cases for the overall image ratings 2–5 can be seen in Supplemental Figure S1.

### 2.6. Statistical Analysis

All statistical analyses were performed using SPSS v23 (IBM Corporation, Armonk, NY, USA). All data were tested for normal distribution using the Shapiro–Wilk test and reported as mean  ±  standard deviation or median with the interquartile range. Categorical data were reported as absolute frequencies and proportions.

Differences between kernel types and sharpness levels were evaluated using univariate analysis of variance with post hoc testing. The *p*-values < 0.05 were considered significant.

Inter-reader agreement was tested with intraclass correlation coefficients (ICC) using the following interpretation: (<0.2) poor agreement, (0.2–0.4) fair agreement, (0.4–0.6) moderate agreement, (0.6–0.8) substantial agreement, and (>0.8) excellent agreement.

## 3. Results

### 3.1. Study Population and Radiation Dose

A total of 30 patients were included in this study. Relevant CAD, namely stenosis of the coronary artery < 50%, was excluded in 22 patients (74%). Detailed patient characteristics and radiation doses are provided in Table 1.

### 3.2. Quantitative Image Analysis

**Figure 2:**
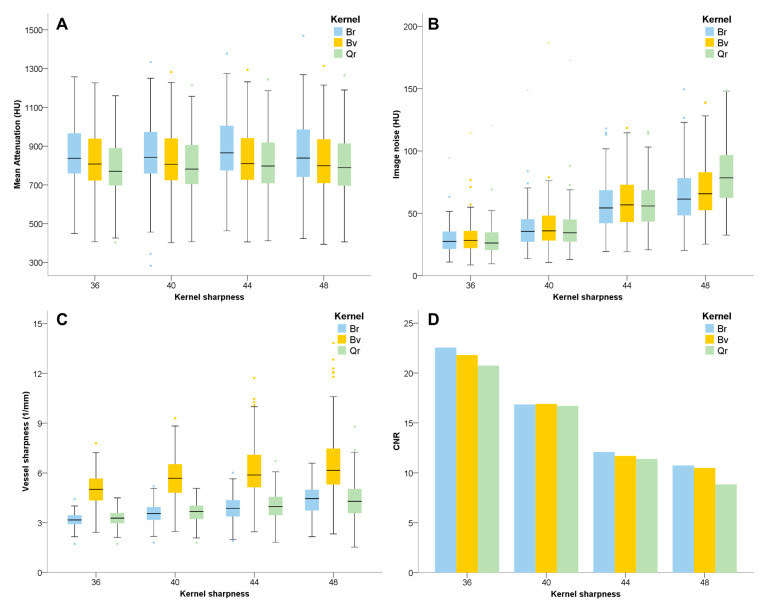
Results of the quantitative image analysis of (**A**) mean attenuation, (**B**) image noise, (**C**) CNR, and (**D**) vessel sharpness on the different reconstruction kernels and sharpness levels.

Detailed results for attenuation, image noise, CNR, and vessel sharpness are provided in Figure 2 and Table 2. Briefly, attenuation, image noise, CNR, and vessel sharpness differed significantly across kernels (for all, *p* < 0.001).

Reconstructions with kernel Br showed the highest mean attenuation compared with kernels Bv (*p* < 0.001) and Qr (*p* < 0.001). Between kernels Bv and Qr, attenuation values were higher in reconstructions with kernel Bv, but significance was only found in the distal coronary artery. Different sharpness level had no relevant influence on attenuation.

Image noise: There was no significant difference on image noise among convolutional kernels. With increasing kernel sharpness, image noise increased significantly.

CNR: In general, reconstruction with kernel Br had the highest CNR, followed by kernels Bv and Qr (Br > Bv > Qr). However, at sharpness level 40, different kernels had similar CNR in the proximal coronary artery, and kernel Bv had a significantly superior CNR in the distal vessel (*p* < 0.001), see Figure S2 in Supplementary Materials. In concordance with image noise, CNR decreased continuously with increasing kernel sharpness levels.

Vessel sharpness: Kernel Bv had significantly higher vessel sharpness levels compared with kernels Br and Qr (*p* < 0.001), whereas no relevant difference was found between kernels Br and Qr (*p* = 0.398). Similar to image noise, vessel sharpness increased significantly with increasing kernel sharpness levels. Furthermore, vessel sharpness between the proximal and distal coronary artery differed significantly in kernels Br48, Qr44, and Qr 48, but no significant difference was observed with kernel Bv, regardless of sharpness level.

Inter-reader agreement was excellent for attenuations (ICC = 0.98), substantial for image noise measurements (ICC = 0.76), excellent for CNR (ICC = 0.99), and moderate for vessel sharpness assessment (ICC = 0.63).

### 3.3. Qualitative Image Analysis

**Figure 3:**
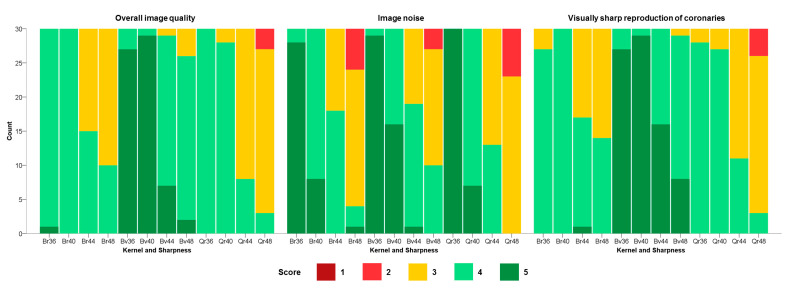
Results of the qualitative image analysis of subjective overall image quality, subjective image noise, and visually sharp reproduction of coronaries using 5-point Likert scales.

Detailed results of the subjective image quality are given in Table 3 and Table 4 and Figure 3.

Overall image quality was rated significantly higher for kernel Bv than for kernels Br and Qr. Among different kernel sharpness levels, sharpness levels 36 and 40 were both rated higher than levels 44 and 48. No significant difference was found among different convolutional kernels. Similar to the objective analysis, subjective image noise increased with increasing kernel sharpness. Therefore, subjective image noise was rated with the best score at sharpness level 36 (36 > 40 > 44 > 48).
Significant differences (*p* < 0.001) were found among different convolutional kernels und sharpness levels regarding visually sharp reproduction of coronaries. Reconstructions with kernel Bv were rated better than reconstructions with kernels Br and Qr. Sharpness levels 36 and 40 achieved better results than levels 44 and 48.
The inter-reader agreement was excellent for overall subjective image quality (ICC = 0.95), subjective image noise (ICC = 0.91), and visually sharp reproduction of coronaries (ICC = 0.98).

## 4. Discussion

This in vivo study evaluated objective and subjective image quality in different reconstruction kernels and sharpness levels for high-pitch, spectral CCTA using a PCD in a clinical setting. The major results were as follows. (1) Attenuation, image noise, CNR, and vessel sharpness differed significantly across kernels, with kernel Br reaching the highest mean attenuation. (2) With increasing kernel sharpness, image noise and vessel sharpness increased, whereas CNR continuously decreased. (3) Reconstruction with kernel Br had generally the highest CNR (Br > Bv > Qr), but a superior CNR was found for kernel Bv at sharpness level 40. (4) Kernel Bv had significantly higher vessel sharpness than kernels Br and Qr. (5) Image quality was subjectively rated best for Bv40 and Bv36, followed by Br36 and Qr36. Based on the results of the objective and subjective image quality analysis, we would recommend the usage of kernel Bv at strength level 40 for image reconstruction of spectral PCD-CCTA in high-pitch Flash mode.

Due to its excellent sensitivity and negative predictive value for the diagnosis and exclusion of coronary artery stenosis, CCTA has become the first-line test for the assessment of patients with suspected CAD, particularly in patients with a low-to-intermediate risk of CAD [4,5]. In addition, compared with invasive coronary angiography (ICA) as the gold standard in the diagnosis of CAD, CCTA avoids the risks associated with an invasive procedure and provides a more expeditious and cost-effective means with less radiation exposure to assess patients with an intermediate risk of CAD [6,21].

However, as coronary arteries are moving structures with typically small diameter (i.e., millimeters), CCTA requires optimal image quality to be interpretable with the highest confidence. In addition to relevant developments in temporal resolution (e.g., by dual-source CT imaging [22]), several limitations of conventional EID-CT remain. The recently introduced PCD-CT enables significantly improved CCTA images for evaluation of calcifications, stents, and noncalcified plaques [17]. A recently published multicenter study also found high image quality in PCD-CCTA, with 95% accessibility of coronary segments and excellent diagnostic performance (sensitivity 92%, specificity 96%) for coronary arteries with low and moderate calcifications [23]. In the presence of heavy calcifications, PCD-CT also provided better visualization of calcium plaques and the patent lumen than EID-CT and more accurate quantification of stenosis in a phantom study [24].

Being a recently introduced technology, little literature is currently available concerning image quality in PCD-CCTA, especially in regard to optimization of the reconstruction parameter. To the best of our knowledge, no previous study has systematically assessed the impact of convolution kernel and kernel sharpness level on image quality in PCD-CCTA with high-pitch Flash mode. In a recently published study, Sartoretti et al. investigated the impact of keV and QIR on image quality in PCD-CCTA [25]. In this phantom and in vivo study, superior image quality was found in 40 keV VMI reconstructions at QIR levels 3 and 4. PCD-CCTA was performed in their study with sequential, not high-pitch, scan mode, and the reconstruction was with a single medium soft convolution kernel. Mergen et al. [16] reported the influence of kernel sharpness on image quality in 20 patients with a high coronary calcium load undergoing ultra-high-resolution PCD-CCTA with sequential gating. In their study, the objective image quality parameter differed significantly across reconstructions. With higher kernel sharpness, CNR continuously decreased, whereas image noise and vessel sharpness increased. Similar results were achieved in our study using high-pitch CCTA acquisitions, but with lower kernel sharpness levels. In the objective image quality analysis, reconstruction with kernels Bv44 and Bv56 were rated as best. This result seemed to be different with our analysis, but an important difference between the studies should be considered. In their study, instead of spectral CCTA in high-pitch Flash mode, they assessed ultra-high-resolution CCTA using retrospectively ECG-gated helical mode. In addition, Mergen et al. only investigated convolution kernel Bv.

The above-mentioned study investigated image characterization in sequential or ultra-high-resolution PCD-CCTA, whereas the present study analyzed image quality in spectral high-pitch PCD-CCTA with different reconstruction kernels and sharpness levels. Our results indicate that reconstruction with kernel Bv40 may be the ideal parameter for spectral PCD-CCTA in high-pitch mode using QIR at strength level 3. This may be clinically relevant, as CCTA is the first-line test for the assessment of patients with suspected CAD with low and intermediate risks. Thus, most patients undergoing CCTA are patients without relevant stenosis and do not need invasive interventions [26]. Therefore, a scan mode with comparably satisfied image quality and lower radiation dose is particularly important. Compared with acquisition using ultra-high-resolution mode [16,17], which most likely suffers from lower z-coverage and lack of spectral information or other scan modes (i.e., prospective ECG-triggered sequence mode and retrospective ECG-gated helical mode), PCD-CCTA with high-pitch Flash mode provides good image quality with a significantly lower radiation dose and simultaneous spectral information [27,28], especially for patients with low plaque burden. With increasing image quality, possibly due to higher temporal resolution in the third-generation dual-source CT [29] and higher photon efficiency of PCD [9], the threshold above which HR starts impacting diagnostic quality is also increased in CCTA with high-pitch Flash mode [30,31,32]. Clinically, results from the aforementioned studies could lead to the generation of dedicated PCD-CCTA protocols depending on the patient’s plaque burden. Though patients with low or no plaque burden (e.g., by coronary artery calcium scoring) can be successfully imaged using a low radiation dose and sufficient image quality using high-pitch CCTA, patients with a relevant burden of coronary artery disease could be imaged with dedicated ultra-high-resolution protocols.

Reconstructions with kernels Br36 and Qr36 also achieved excellent image quality in the overall subjective image quality and CNR. In addition to the regularly used vascular kernels (i.e., Bv) for the assessment of vessels, the body regular kernel (Br) is needed for soft tissue evaluation, and the quantitative kernel (Qr) is the basis for post-processing of spectral image information, which represents a key, unique feature of PCD-CT [14]. Based on these spectral data, high-quality virtual non-iodine (VNI) or virtual non-calcium (VNCa) images can be reconstructed, allowing calcium scoring without true non-contrast (TNC) acquisition [33] and counteracting the problem of blooming artifacts from heavy calcified plaques [34]. Therefore, our results would also be useful for evaluating other cardiac structures or assessing spectral quantitative images with optimal image quality.

The following limitations merit consideration. First, this study only included a limited number of patients who underwent PCD-CCTA to exclude or assess CAD at a single center. This prohibited us from analyzing image quality in patients with known CAD and therefore also those with coronary stents. Additionally, the distribution of CAD-RADS classes was not balanced, with CAD-RADS 0 being the predominant group. Despite quantitative and qualitative image measures independent from CAD-RADS class, a more balanced distribution could further improve the generalizability of the results. Second, only variations in different kernel types and sharpness levels were evaluated in this study; the effects of variation of other reconstruction parameters, such as QIR or keV levels, should be considered in future studies. Third, the effect of kernel and kernel sharpness on stenosis quantification and plaque analysis was not investigated in this study. In this regard, further studies leading to clinical conclusions should be performed. Finally, all scans were performed in high-pitch Flash mode with spectral images. Optimal kernel and sharpness were not assessed for newly developed ultra-high-resolution PCD-CCTA with non-spectral data or other scan modes.

## 5. Conclusions

The use of different kernels and strength levels influences the subjective and objective image quality of spectral, high-pitch PCD-CCTA examinations. Reconstructions with kernel Bv at strength level 40 seem to be beneficial to achieve optimal image quality. Effects on clinical decision-making, such as plaque quantification and stenosis/CAD-RADS grading, have to be determined in future studies.

## Figures and Tables

**Table 1:** Patient characteristics and radiation dose.

Patient Characteristics (*n* = 30)	
Age, years	63 ± 12.6 (range 39–83)
Female	8 (27%)
Body mass index, kg/m²	25.4 ± 2.98
Heart rate, bpm	59 ± 7.1 (range 37–70)
CAD-RADS	
0	16 (54%)
1	4 (13%)
2	2 (7%)
3	1 (3%)
4	6 (20%)
N	1 (3%)
Radiation dose	
CTDI, mGy	2.68 ± 0.59
DLP, mGy·cm	53.51 ± 8.91
Effective dose, mSv	1.07 ± 0.18

Values are given as mean ± standard deviation or *n* (%) unless otherwise noted. bpm: beats per minute; CAD-RADS: Coronary Artery Disease—Reporting and Data System; CTDI: computed tomography dose index; DLP: dose length product.

**Table 2:** Results of quantitative image analysis.

Kernel		Attenuation *	Noise ^ꝉ^	Sharpness ^#^	CNR
Br	36	863.3 ± 161.1	37.9 ± 8.8	3.2 ± 0.4	22.6 ± 0.9
	40	863.7 ± 171.5	50.5 ± 14.8	3.9 ± 0.8	16.9 ± 0.5
	44	889.8 ± 168.3	72.4 ± 14.9	3.9 ± 0.8	12.1 ± 0.3
	48	861.0 ± 174.3	79.3 ± 14.4	4.4 ± 0.9	10.8 ± 0.1
Bv	36	832.6 ± 156.6	38.0 ± 9.1	5.1 ± 1.0	21.8 ± 0.8
	40	832.9 ± 159.5	48.8 ± 10.4	5.8 ± 1.2	16.9 ± 0.4
	44	832.1 ± 162.4	71.3 ± 14.6	6.5 ± 3.8	11.7 ± 0.3
	48	822.7 ± 165.0	78.1 ± 14.1	6.9 ± 3.9	10.5 ± 0.3
Qr	36	730.0 ± 149.5	37.4 ± 8.3	3.3 ± 0.5	20.8 ± 1.1
	40	805.2 ± 152.7	48.0 ± 9.6	3.7 ± 0.6	16.7 ± 0.7
	44	815.5 ± 157.4	70.6 ± 14.0	4.0 ± 0.9	11.4 ± 0.4
	48	810.8 ± 161.9	90.8 ±16.9	4.4 ± 1.2	8.9 ± 0.4

* Mean coronary attenuation in Hounsfield units. ^ꝉ^ Mean image noise in Hounsfield units measured in adipose tissue adjacent to the right coronary artery. ^#^ Edge sharpness measured as slope of the attenuation change curve (1/mm).

**Table 3:** Influence of different reconstruction kernels on subjective image analysis.

		Br	Bv	Qr	*p*-Value
Subjective overall image quality	All raters	4 (3–4)	5 (4–5)	4 (3–4)	<0.001
	Rater 1	4 (3–4)	5 (4–5)	4 (3–4)	<0.001
	Rater 2	4 (3–4)	5 (4–5)	4 (3–4)	<0.001
Image noise	All raters	4 (3–5)	4 (3–5)	4 (3–5)	0.12
	Rater 1	4 (3–5)	4 (3–5)	4 (3–5)	0.22
	Rater 2	4 (3–5)	4 (3–5)	4 (3–5)	0.10
Visually sharp reproduction of coronaries	All raters	4 (3–4)	5 (4–5)	4 (3–4)	<0.001
	Rater 1	4 (3–4)	5 (4–5)	4 (3–4)	<0.001
	Rater 2	4 (3–4)	5 (4–5)	4 (3–4)	<0.001

**Table 4:** Influence of kernel sharpness level on subjective image analysis.

		36	40	44	48	*p*-Value
Subjective overall image quality	All raters	4 (4–5)	4 (4–5)	4 (3–4)	4 (3–4)	<0.001
	Rater 1	4 (4–5)	4 (4–5)	4 (3–4)	4 (3–4)	<0.001
	Rater 2	4 (4–5)	4 (4–5)	4 (3–4)	4 (3–4)	<0.001
Image noise	All raters	5 (5–5)	4 (4–5)	4 (3–4)	3 (3–3)	<0.001
	Rater 1	5 (5–5)	4 (4–5)	4 (3–4)	3 (3–3)	<0.001
	Rater 2	5 (5–5)	4 (4–5)	4 (3–4)	3 (3–3)	<0.001
Visually sharp reproduction of coronaries	All raters	4 (4–5)	4 (4–5)	4 (3–4)	4 (3–4)	<0.001
	Rater 1	4 (4–5)	4 (4–5)	4 (3–4)	4 (3–4)	<0.001
	Rater 2	4 (4–5)	4 (4–5)	4 (3–4)	4 (3–4)	<0.001

Ratings in both tables are presented as median (IQR). All *p*-values are derived from independent-sample Kruskal–Wallis tests.

## Data Availability

The datasets analyzed during the current study are available from the corresponding author on reasonable request.

## Supplementary Materials

The following supporting information can be downloaded at https://www.mdpi.com/article/10.3390/diagnostics13111937/s1. Figure S1: Exemplary images with overall image ratings of 2 (tile A), 3 (tile B), 4 (tile C), and 5 (tile D) from the same patient. Figure S2: Results of the quantitative image analysis split for proximal (left) and distal (right) vessels with CNR (top) and Vessel sharpness (bottom). Table S1: Objective image quality in proximal and distal vessels.
