# Supplementary material for: Optimization of Kernel Type and Sharpness Level Improves Objective and Subjective Image Quality for High-Pitch Photon Counting Coronary CT Angiography
Source: Diagnostics (Basel). 2023 Jun 1;13(11):1937. doi: 10.3390/diagnostics13111937 (PMC10252999; doi:10.3390/diagnostics13111937)
Supplement: Supplementary file 1 [file diagnostics-13-01937-s001.zip › Supplemental Table S1.pdf]

Supplemental Table S1: Objective image quality in proximal and distal vessels

Kernel		Proximal vessels			Distal vessels		
		Attenuation	CNR	Edge	Attenuation	CNR	Edge
Br	36	882 + 164	23.2	3.07	834 + 154	21.9	3.29
	40	878 + 173	17.2	3.45	840 + 170	16.5	3.67
	44	900 + 171	12.3	3.81	872 + 169	11.9	4.00
	48	864 + 170	10.8	4.41	853 + 185	10.7	4.44
Bv	36	850 + 160	22.4	5.05	807 + 152	21.2	5.09
	40	845 + 161	17.2	5.70	814 + 160	16.6	5.79
	44	845 + 163	11.9	6.03	813 + 167	11.5	6.18
	48	834 + 163	10.7	6.43	805 + 171	10.3	6.49
Qr	36	816 + 151	21.5	3.19	759 + 143	20.0	3.39
	40	825 + 154	17.2	3.55	776 + 150	16.2	3.77
	44	834 + 158	11.7	3.92	788 + 157	11.1	4.13
	48	828 + 162	9.1	4.27	785 + 165	8.6	4.46
