# Supplementary figures and images for: Optimization of Kernel Type and Sharpness Level Improves Objective and Subjective Image Quality for High-Pitch Photon Counting Coronary CT Angiography
Source: Diagnostics (Basel). 2023 Jun 1;13(11):1937. doi: 10.3390/diagnostics13111937 (PMC10252999; doi:10.3390/diagnostics13111937)

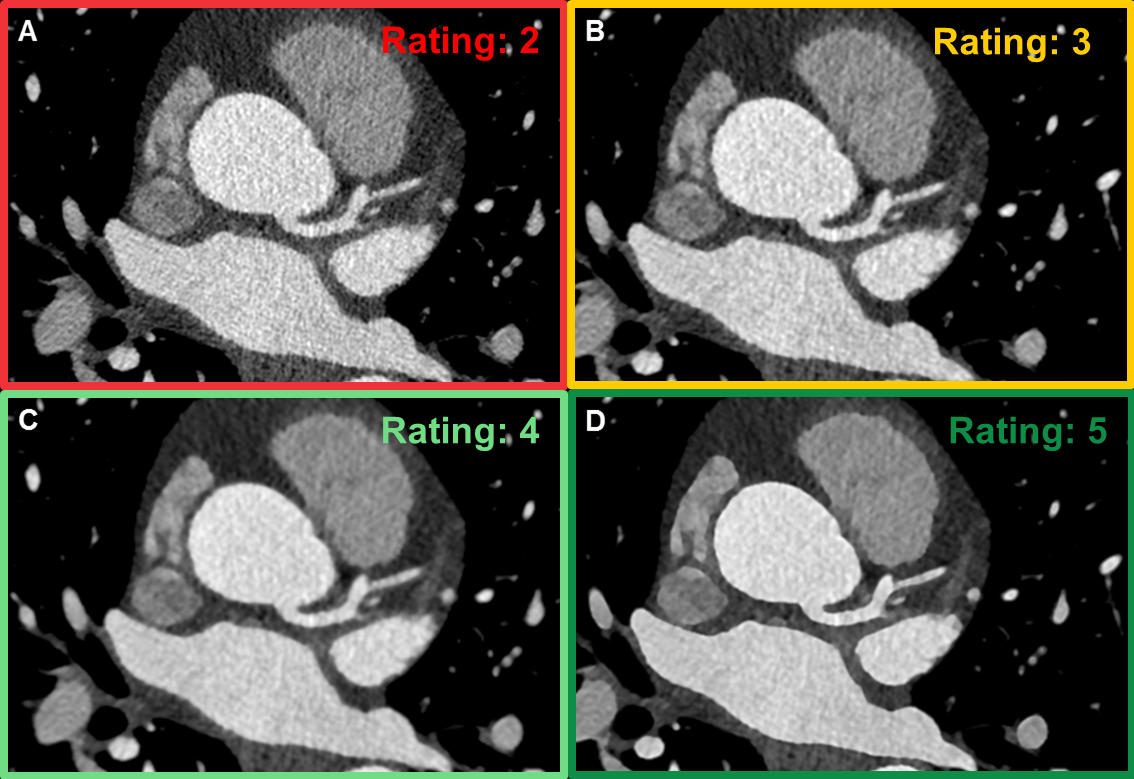

Supplement: Supplementary file 1 [file diagnostics-13-01937-s001.zip › Supplemental Figure S1.tif]

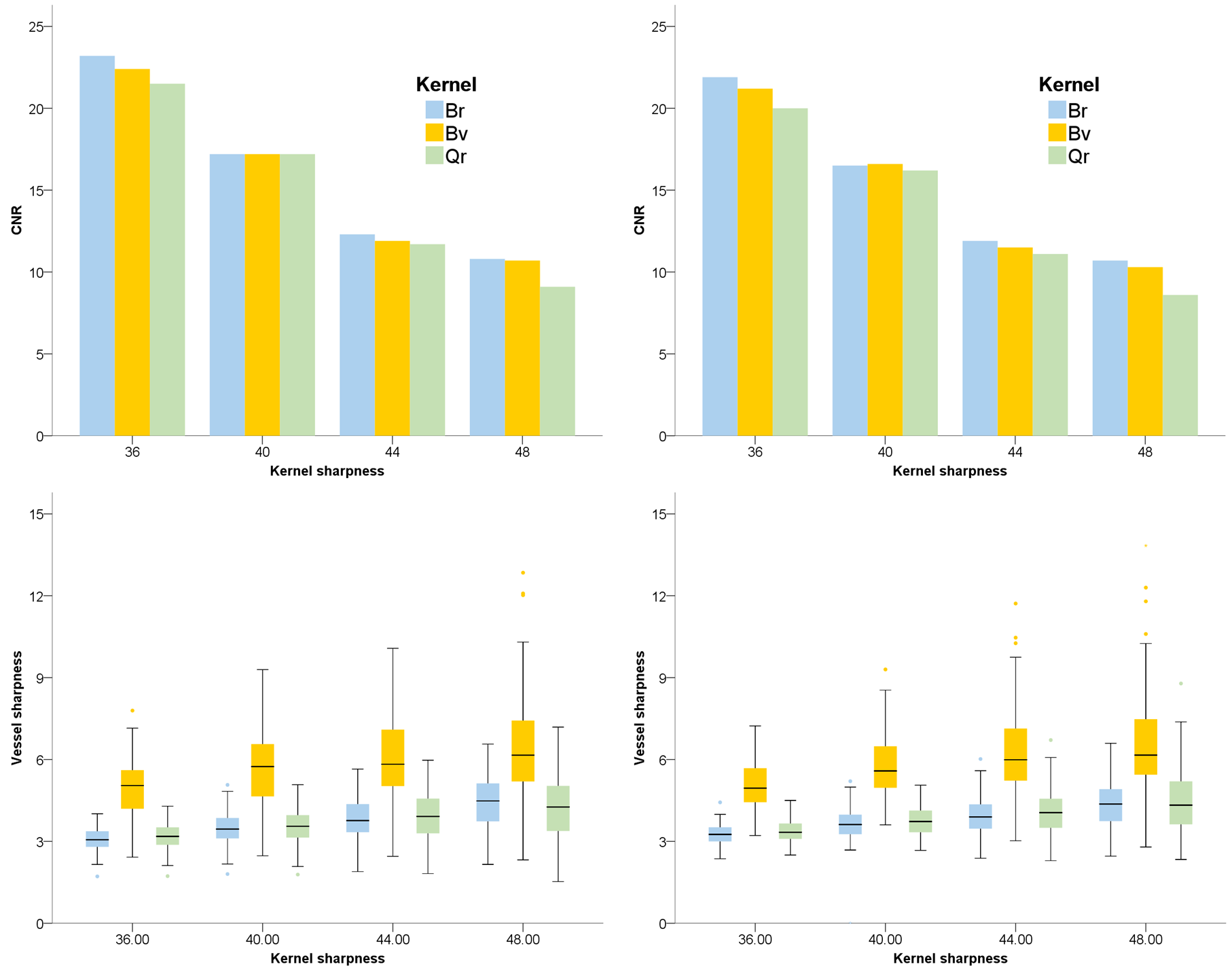

Supplement: Supplementary file 1 [file diagnostics-13-01937-s001.zip › Supplemental Figure S2.tif]
